# Impact of *Mycobacterium tuberculosis* RD1-locus on human primary dendritic cell immune functions

**DOI:** 10.1038/srep17078

**Published:** 2015-11-25

**Authors:** Marilena P. Etna, Elena Giacomini, Manuela Pardini, Martina Severa, Daria Bottai, Melania Cruciani, Fabiana Rizzo, Raffaele Calogero, Roland Brosch, Eliana M. Coccia

**Affiliations:** 1 Department of Infectious, Parasitic and Immune-mediated Diseases; Istituto Superiore di Sanità; Rome, Italy; 2 Department of Translational Research and new Technologies in Medicine and Surgery, University of Pisa; Pisa, Italy; 3 Institut Pasteur, Unit for Integrated Mycobacterial Pathogenomics; Paris, France; 4 Molecular Biotechnology Center, Department of Molecular Biotechnology and Health Sciences; University of Torino; Turin, Italy

## Abstract

Modern strategies to develop vaccines against *Mycobacterium tuberculosis* (Mtb) aim to improve the current Bacillus Calmette-Guerin (BCG) vaccine or to attenuate the virulence of Mtb vaccine candidates. In the present study, the impact of wild type or mutated region of difference 1 (RD1) variants on the immunogenicity of Mtb and BCG recombinants was investigated in human primary dendritic cells (DC). A comparative analysis of transcriptome, signalling pathway activation, maturation, apoptosis, cytokine production and capacity to promote Th1 responses demonstrated that DC sense quantitative and qualitative differences in the expression of RD1-encoded factors—ESAT6 and CFP10—within BCG or Mtb backgrounds. Expansion of IFN-γ producing T cells was promoted by BCG::RD1-challenged DC, as compared to their BCG-infected counterparts. Although Mtb recombinants acted as a strong Th-1 promoting stimulus, even with RD1 deletion, the attenuated Mtb strain carrying a C-terminus truncated ESAT-6 elicited a robust Th1 promoting phenotype in DC. Collectively, these studies indicate a necessary but not sufficient role for the RD1 locus in promoting DC immune-regulatory functions. Additional mycobacterial factors are likely required to endow DC with a high Th1 polarizing capacity, a desirable attribute for a successful control of Mtb infection.

Tuberculosis (TB) remains one of the principal leading causes of death worldwide accounting for 9 million new cases and about 1.5 million deaths each year^1^. The attenuated *Mycobacterium bovis*—*bacillus Calmette-Guerin* (BCG)—is the only currently available vaccine targeted against TB. Because BCG provides limited and variable efficacy against the pulmonary form of the disease and has an unknown impact on protection of latently infected individuals, there is a urgent unmet need to develop new TB vaccines^2^. Novel genomic technologies have identified significant differences between *Mycobacterium tuberculosis* (Mtb) and BCG genomes that have furthered the development of numerous live attenuated recombinant strains with vaccine potential. The main genetic modification involved in the attenuation of the vaccine strain BCG is the deletion of the so-called genomic region of difference 1 (RD1)^3^, which is required for full virulence of Mtb. Indeed, the RD1 encodes two strong immunogenic antigens and virulence factors—the 6-kDa early secreted antigenic target (ESAT-6) and the 10-kDa culture filtrate protein (CFP-10)^4^—as well as several structural components of the ESAT-6 secretion system (ESX)-1 type VII secretion system, which is responsible for ESAT-6 and CFP-10 secretion^5^,^6^.

Several TB vaccination strategies are based either on improvements in BCG immunogenicity or on attenuation of Mtb virulence through insertion, mutation or deletion of this locus. When integrated into the BCG genome, the extended RD1 region improved the ability of BCG to protect against dissemination of TB in mouse and guinea pig models^7^. On the other hand, one of the most promising Mtb-based live vaccines (MTBVAC, NCT 02013245), which is currently in clinical trial, does not secrete the RD1-encoded factors and has acquired an attenuated phenotype without impairment of the immunogenic potential^8^. The importance of the RD1 region in TB immunopathology resides in the crucial role that RD1-encoded factors play in controlling host-pathogen interactions. For instance, in macrophages, RD1- encoded molecules rupture the phagosomal membrane-bound compartment allowing the bacterium to get access to the host cytosol. Bacterial products sensed inside the cytosol elicit cell death^9^,^10^,^11^,^12^, promote inflammasome activation with the related production of interleukin (IL)-1β and IL-18^13^,^14^ and, at the same time, become target of cytosolic components of the ubiquitin-mediated autophagy pathway^15^. A more complex link between RD1 and the control of the autophagic flux was recently identified by our group in human dendritic cells (DC)^16^. In this setting, we demonstrated that the autophagy block induced by RD1-encoded ESX-1 secretion system was overcome by a treatment with the autophagy inducer, rapamycin, which further induced IL-12 production and, in turn, strengthened the capacity of DC to expand a T helper (Th)1-oriented response.

Given the central role of human DC in orchestrating vaccine-induced immunity, an understanding of the DC-specific innate responses triggered by Mtb or BCG vaccine candidates will be critical for the design of efficient vaccine strategies. Thus, we sought to investigate how infection with BCG and Mtb recombinant strains, complemented with or deleted in the RD1 locus, or expressing mutated variants of the ESAT-6/CFP-10 complex, would impact DC functions. These studies revealed the capacity of DC to sense quantitative and qualitative differences among the Mtb and BCG recombinants, and highlight a necessary but not sufficient role for the RD1 locus in promoting DC immune-regulatory functions.

## Results

### Transcriptome analysis of DC challenged with BCG and Mtb recombinant strains

**Figure 1:**
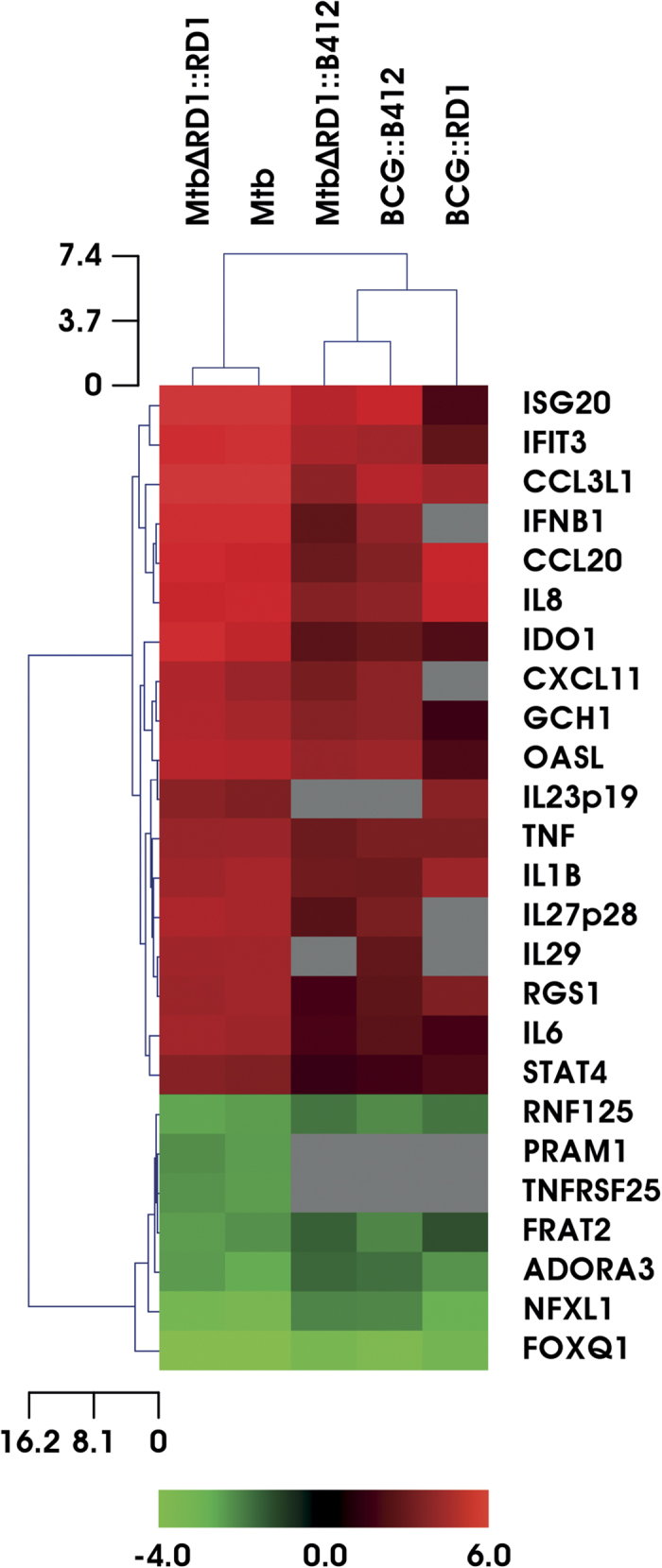
Transcriptome analysis of DC challenged with BCG and Mtb recombinants. Hierarchical clustering of genes differentially expressed in DC challenged for 8 h with parental Mtb, MtbΔRD1::RD1, MtbΔRD1::B412, BCG::RD1 or BCG::B412. The values in grey indicate that the expression of the gene was not significantly different in respect to not infected cells (Ctrl).

**Figure 2:**
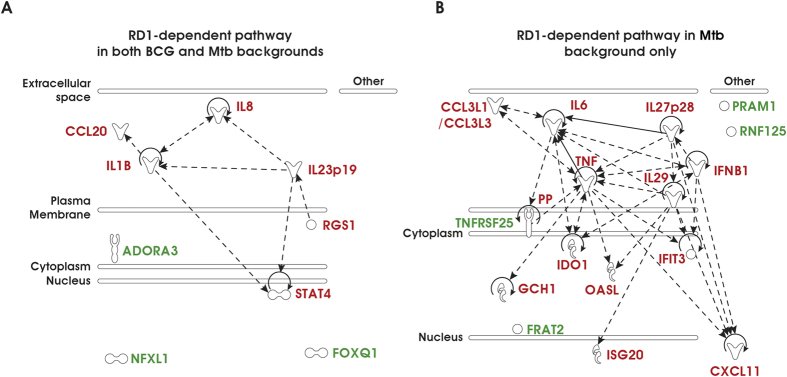
Transcriptional signature of DC challenged with BCG and Mtb recombinants. Transcriptional signature of DC arranged by using Ingenuity Pathway Analysis for the genes modulated by RD1 locus in both BCG and Mtb backgrounds (**A**) or induced by RD1 region expressed within a Mtb-background only (**B**). Induced genes are represented in red, while repressed ones are shown in green.

To investigate whether the RD1 locus and specifically the ESX-1 secretion system may impact the global gene expression profile in DC, a comparative microarray analysis was performed in primary human DC infected with parental Mtb or Mtb and BCG recombinant strains, expressing the RD1 region (MtbΔRD1::RD1 and BCG::RD1, respectively) or lacking the RD1 genetic locus (MtbΔRD1::B412 and BCG::B412, respectively) (for further details on strain characteristics and virulence see Supplementary Table S1). Total RNA was isolated, hybridized on the HumanHT-12 v4 Expression BeadChips (Illumina) and analyzed with the oneChannelGUI Bioconductor package^17^. Among the significantly expressed genes, the top up- and down-regulated transcripts with a minimum fold change of 2.5 were selected for further analysis (Fig. 1). Data set analysis revealed that DC infection with either wild type (wt) Mtb or MtbΔRD1::RD1 (recombinant strain carrying the extended RD1 locus as the parental Mtb) up-regulated the transcription of 215 genes in DC, whereas infection with MtbΔRD1::B412 (recombinant strain deleted in the RD1 region) up-regulated ~100 genes. Conversely, BCG::RD1 (recombinant strain containing the extended RD1 region) and BCG::B412 (recombinant strain containing the empty vector pYUB412) induced the expression of a lower number of genes (~60) as compared to Mtb strains; also a small number of transcripts (~20) was found to be down-regulated in all experimental conditions. The differentially expressed genes were associated primarily with three pathways: immune cell trafficking, inflammatory response and IFN signaling (Fig. 2). Specific RD1-dependent gene signatures, common to both Mtb and BCG strains or specifically regulated only by Mtb, were identified and a hierarchical clustering analysis of this gene set is shown in Fig. 1. Transcripts that were RD1-regulated in both bacterial backgrounds were predominantly associated with inflammatory and immune cell trafficking pathways (Table 1A), including: IL1B, an important mediator of the inflammatory response; chemokine C-C motif ligand 20 (CCL20), a chemotactic factor that attracts immature DC with a role in mucosal immunity against Mtb and other bacteria^18^; signal transducer and activator of transcription-4 (STAT4), a transcription factor induced by inflammatory stimuli and many pathogens contributing to the Th1 polarization^19^; CXC family ligand-8 (CXCL8, also called IL-8), a chemokine involved in the recruitment of neutrophils and T cells in the lung during Mtb infection^20^ and regulator of G-protein signaling 1 (RGS1), a member of the G-protein-coupled receptor family principally regulating chemokine and chemokine receptors and, in turn, orchestrating immune cell trafficking during responses to exogenous or infectious agents^21^,^22^. Interestingly, among the cellular genes induced in response to Mtb and BCG recombinant strains carrying RD1, the induction of IL23p19, which might cooperate with IL-1β and other cytokines in driving the Th17 response, was also observed^23^,^24^ (see below for further details).

With regard to the specific Mtb-induced RD1-dependent specific gene signature, transcripts belonging to type I IFN and inflammation-related signaling were over-represented (Table 1B), indicating that Mtb infection of DC modulated the amplitude of the inflammatory responses compared to BCG, specifically inducing numerous well characterized type I IFN-stimulated genes (ISGs), including ISG20, IFN-induced protein with tetratricopeptide repeats 3 (IFIT3), 2′-5′-oligoadenylate synthetase-like protein (OASL), indolemine 2,3 dioxygenase-1 (IDO1), IL27p28, CXCL11, and pro-inflammatory cytokines such as tumor necrosis factor (TNF)-α, IL29 and IL6.

**Figure 3:**
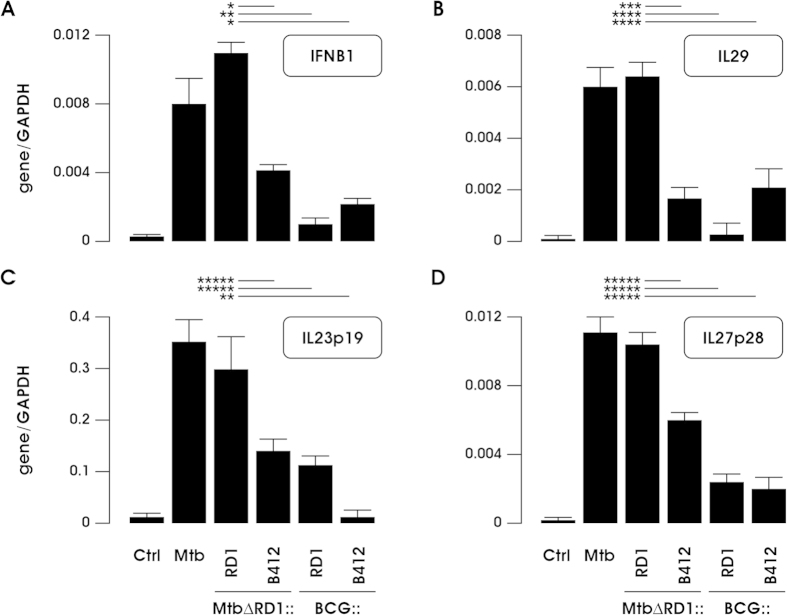
Validation of transcriptome data on IFNB1, IL29, IL23p19 and IL27p28 gene expression. Real-time RT-PCR analysis was conducted in untreated DC (Ctrl) or in DC infected for 8 h with wt Mtb or with the indicated Mtb and BCG strains to evaluate the expression of IFNB1 (A), IL29 (B), IL23p19 (C) and IL27p28 (D). All quantification data are normalized to the GAPDH level using the equation 2^−ΔCt^. For real-time RT-PCR, the shown results were mean of 4 experiments performed with RNAs derived from a set of experiments independent than those used in the transcriptome studies. p-values: ^*^≤0.03; ^**^≤0.02; ^***^≤0.004; ^****^≤0.001; ^*****^≤0.04.

To validate the transcriptome analysis data, quantitative real time PCR experiments were conducted in DC infected with Mtb and BCG recombinants. A robust induction of IFNB1 and IL29 (also known as IFNL1) was observed in DC infected with either wt Mtb or MtbΔRD1::RD1 (Fig. 3) and correlated with the gene expression data demonstrating ISG up-regulation (Fig. 2 and Table 1B). Interestingly, the levels of IL23p19 and IL27p28, two subunits of IL-12 family members, were also strongly induced in response to Mtb expressing RD1-encoded factors, whereas RD1 inserted in the BCG backbone stimulated the expression of IL23p19 only, thus confirming the results of gene expression profiling (Figs 1, 2, 3 and Table 1A). This observation prompted us to extend our analysis to other subunits of the heterodimeric cytokines IL-23 and IL-27: IL12B (also known as IL12p40) that pairs with IL23p19 to constitute IL-23, and EBI3 that associates with IL27p28 to form IL-27^25^. Expression of both IL12p40 and EBI3 subunits was induced by RD1 in the Mtb background, as occurs in response to Mtb infection (Supplementary Fig. S1). In contrast, BCG reconstituted with RD1 induced the expression of IL12p40, whereas the EBI3 transcript was reduced in DC infected with BCG:: RD1(Supplementary Fig. S1).

**Figure 4:**
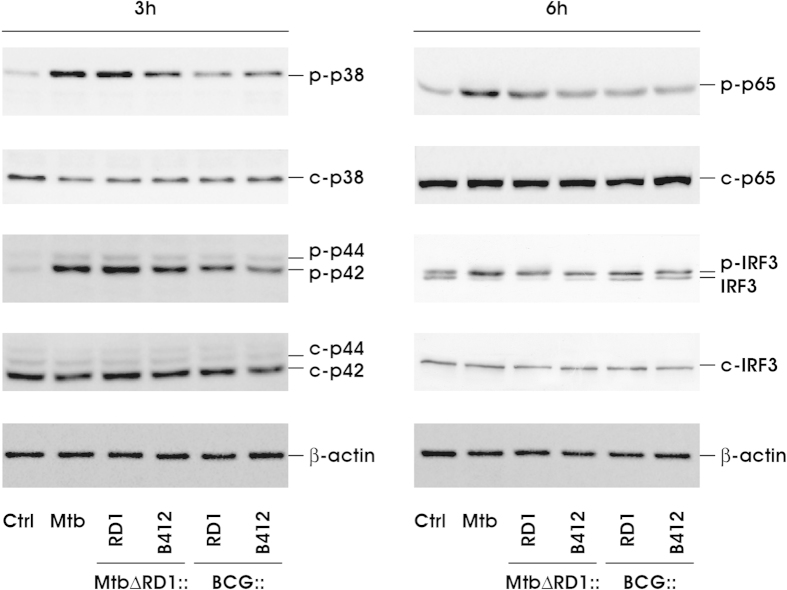
Analysis of p38, p44/42 MAPK, NFKB p65 and IRF3 activation. The intracellular pathways involving the activations of p38, p44/42 MAPK (3 h post infection) and of NFKB p65 subunit, IRF3 transcriptional factor (6 h post infection) were investigated by western blotting on whole cell extracts prepared from DC infected with wt Mtb or with the indicated Mtb and BCG strains. To detect the phosphorylated IRF3 isoforms (p-IRF3, upper panel), cell extracts were analyzed on an SDS-10% PAGE gel and subjected to immunoblot analysis with anti-IRF3 Ab, while a shorter electrophoretic run on an SDS-10% PAGE gel was performed to analyze the relative levels of IRF3 content (lower panel). A representative experiment, out of 3 independent experiments performed, is shown.

Providing that RD1-encoded factors were shown to influence the signaling pathways leading to cytokine gene expression in Mtb infected cells^26^, we investigated in DC challenged with Mtb and BCG recombinants the intracellular activation of Mitogen-activated protein kinase (MAPK) and Nuclear Factor (NF)-kB, both involved in the transcriptional regulation of TNF and IL-12 family members (Fig. 4). A robust phosphorylation of MAPK, namely p38 and p44/42 (ERK1/2), was observed 3 h after infection with both wt Mtb or MtbΔRD1::RD1 as compared to MtbΔRD1::B412, which displayed a lower capacity to stimulate their activation. A fainter level of MAPK phosphorylated forms was found in total cell extracts derived from both BCG::RD1 and BCG::B412 infected DC. This activation profile mirrors that of the down-stream NF-kB p65 phosphorylation. In addition, the phosphorylation of IFN regulatory factor 3 (IRF3), master regulator of type I IFN and IL29 gene expression^27^,^28^, was also analyzed (Fig. 4). Infection with wt Mtb, MtbΔRD1::RD1 and, slightly, MtbΔRD1::B412, stimulated IRF3 phosphorylation, while minor modifications were detected in DC infected with BCG recombinants providing evidences for the IRF3-dependent transcriptional induction of IFNB1 and IL29 genes shown in Fig. 3.

Collectively, these data demonstrate that the RD1 locus confers to Mtb and BCG a differential capacity to modulate the expression of IL-12 and IFN family members.

### Different induction of pro-inflammatory cytokines in DC infected with BCG and Mtb recombinants

**Figure 5:**
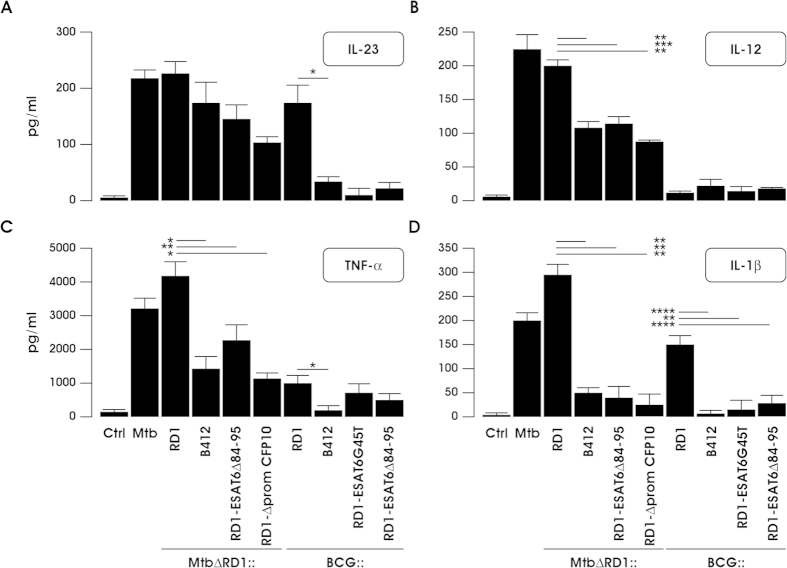
BCG and Mtb RD1-recombinant strains as different inducers of pro-inflammatory cytokines along DC infection. DC were left untreated (Ctrl) or infected for 24 h with wt Mtb or with the indicated Mtb and BCG recombinant strains. IL-23 production was measured by ELISA (A). The secretion of IL-12 (**B**), TNF-α (**C**) and IL-1β (**D**) was instead evaluated by Inflammatory Cytokine kit. The results represent means + SEM of 7 independent experiments. Significance was calculated by Student’s t-test for paired data. p-values: ^*^≤0.05; ^**^≤0.005; ^***^≤0.01; ^****^≤0.0002.

To correlate gene expression profile with secretion data, the presence of pro-inflammatory cytokines was analyzed in DC culture supernatants infected with the Mtb or BCG RD1 recombinants, as well as variants expressing selected mutations in ESAT-6 and CFP-10 (for details about Mtb and BCG recombinants see Supplementary Table S1). As predicted by the gene expression data, the reintroduction of the entire RD1 locus into the BCG background conferred to infected-DC the ability to release IL-23 (Fig. 5A); importantly, IL-23 release was abrogated by a single point mutation in ESAT-6 (BCG::RD1-ESAT6G45T), or by a deletion of 12 amino acids at the ESAT-6 C-terminus (BCG::RD1-ESAT6Δ84-95). In contrast, IL-23 production was sustained in all Mtb-infected cells, regardless of deletion or mutation in RD1 (Fig. 5A).

The analysis of cytokine release was extended to IL-12, which together with IL-23, promotes the expansion of IFN-γ producing cells^29^,^30^,^31^. Strikingly, IL-12 production was not detected in DC infected with BCG or BCG recombinants containing the entire RD1 region or carrying point mutation (BCG::RD1-ESAT6G45T) or deletion (BCG::RD1-ESAT6Δ84-95) in ESAT-6 (Fig. 5B). Conversely, IL-12 was produced by all DC cultures infected with either wt or recombinant Mtb strains. However, the release of IL-12 was higher after infection with Mtb strains containing an intact RD1 region, suggesting that other factors, in addition to RD1, contribute to IL-12 production. When expression of the pro-inflammatory cytokines TNF-α and IL-1β was examined, a clear dependence on RD1 was observed for both BCG and Mtb recombinants (Fig. 5C,D). BCG::RD1 strongly enhanced the capacity of infected DC to release IL-1β, whereas the deletion of the entire RD1 locus or the specific mutation of ESAT-6 and CFP-10 genes impaired both TNF-α and IL-1β secretion, regardless of the bacterial background (Fig. 5C,D). Thus, it is likely that the complex gene regulation driven in DC by RD1 alone or in combination with other mycobacterial factors, might be converted into a specific cytokine milieu controlling the immune response against mycobacteria or recombinant vaccine candidates.

### Effect of Mtb and BCG recombinant strain infection on DC maturation and apoptosis

**Figure 6:**
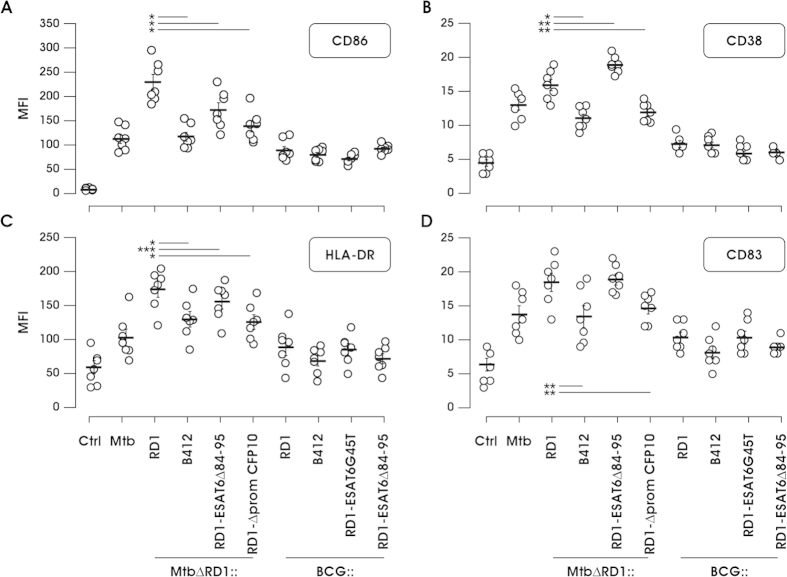
DC maturation in response to infection with Mtb and BCG strains. DC were left untreated (Ctrl) or infected for 24 h with parental Mtb or Mtb and BCG strains engineered in the RD1 locus. Expression of indicated molecules was calculated in 7 independent experiments using the MFI after the subtraction of the values of the isotype Ab controls. MFI value for each individual experiment is shown and data are reported as the MFI mean ± SEM. Statistical significance was determined by Student’s t-test for paired data. p-values: ^*^≤0.00003; ^**^≤0.007; ^***^≤0.003.

Next the capacity of Mtb and BCG RD1-recombinant strains to modulate DC phenotype upon infection was evaluated by flow cytometric measurement of co-stimulatory molecule cluster of differentiation (CD)86, the activation marker CD38, the MHC-II molecule HLA-DR and the maturation marker CD83 (Fig. 6). DC challenge with wt or mutated Mtb strains induced expression of all the above markers, and MtbΔRD1::RD1 generated the most robust maturation profile. Conversely, BCG recombinants also promoted DC maturation, but to a lower extent, suggesting again that the maturation process requires in addition to RD1, other Mtb-specific factors that are not present in BCG.

**Figure 7:**
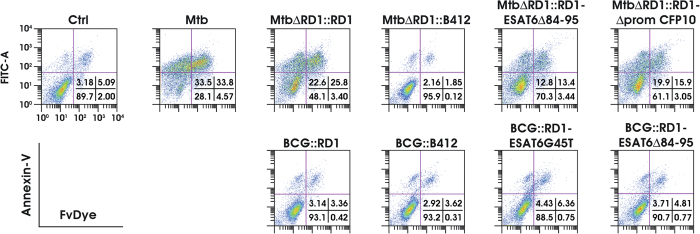
Different impact of BCG and Mtb mutants on DC apoptosis. DC were left untreated (Ctrl), or infected for 24 h with wt Mtb or Mtb and BCG recombinant strains. Apoptosis was assessed by FACS analysis through Annexin-V and FvDye staining. A representative experiment, out of 5 independent experiments performed, is shown. Numbers in the dot plots correspond to the percentage of cells in each quadrant.

We next determined whether infection with Mtb and BCG recombinants affected DC apoptosis (Fig. 7), by labeling DC with Annexin-V in combination with a fixable viability dye (FvDye)^31^. Both wt Mtb and MtbΔRD1::RD1-infected DC displayed a strong apoptotic phenotype as shown by the enhanced percentage of both early (Annexin-V^+^/FvDye^−^) and late apoptotic cells (Annexin-V^+^/FvDye^+^); in contrast, the infection with Mtb lacking RD1 locus did not promote apoptosis in the infected DC (Fig. 7). An intermediate apoptotic phenotype was observed after infection with both MtbΔRD1::RD1-ESAT6Δ84-95 and MtbΔRD1::RD1-ΔpromCFP10 variant strains. In contrast, BCG recombinant strains showed no evidence of enhanced early or late apoptosis (Fig. 7), again indicating that DC apoptosis is regulated by both RD1-encoded factors and, additional as yet unidentified Mtb-encoded factors.

### Differential expansion of Th1 response by DC challenged with Mtb and BCG recombinant strains

**Figure 8:**
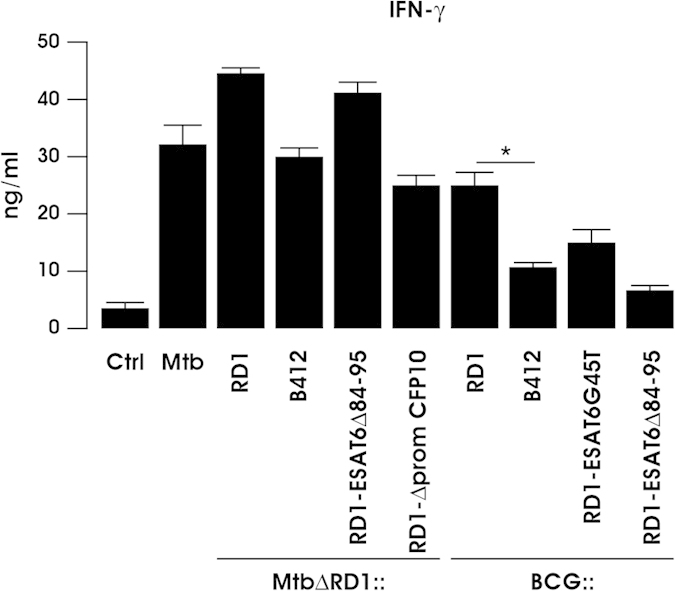
Sufficient but not necessary role of ESX-1 secretion system in eliciting a protective Th1 response. Control DC (Ctrl) and DC challenged for 24 h with the parental Mtb or with the indicated Mtb and BCG mutant strains were co-cultured with allogeneic CD4^+^ T for 5 days. IFN-γ production was evaluated by ELISA in harvested supernatants. The results shown represent means + SEM of 6 independent experiments. Significance was calculated by Student’s t-test for paired data, p-value: ^*^≤0.04.

The capacity of DC infected with wt and recombinant Mtb and BCG strains to stimulate IFN-γ production by allogeneic CD4^+^ T cells was then investigated in a mixed leukocyte reaction (MLR) (Fig. 8). Infection with the BCG::RD1 strain doubled the capacity of DC to expand IFN-γ producing T cells, compared to BCG::B412 or BCG mutants carrying point mutation (BCG::RD1-ESAT6G45T) or deletion (BCG::RD1-ESAT6Δ84-95) in ESAT-6. While a robust IFN-γ production was observed in MLR conducted with DC infected with all Mtb recombinants, the MtbΔRD1::RD1-ESAT6Δ84-95 and MtbΔRD1::RD1 recombinant strains conferred to DC the strongest capacity to promote Th1 phenotype (Fig. 8). This result correlated well with the data on cytokine release, DC maturation and apoptotic phenotype elicited by MtbΔRD1::RD1-ESAT6Δ84-95, and demonstrates that the deletion of 12 amino acids at the C-terminus of ESAT-6 does not affect the capacity of Mtb to activate the immune functions of DC, in spite of a reduced virulence phenotype.

## Discussion

Advances in the understanding of the mechanisms underlying protection against human pathogens are crucial for the development of novel strategies for infection prevention and treatment. How host innate immunity instructs an adaptive immune response that protects against active disease by restricting Mtb replication and transmission in the majority of individuals, remains unknown.

In the present study, we focused on the effects of the Mtb-expressed RD1 locus on DC function, and observed that RD1-encoded factors differentially modulated the host immune response based on the genetic background in which they were expressed (Fig. 2 and Table 1). For example, analysis of DC transcriptome after Mtb infection identified a network of cytokine genes involved in the establishment of the inflammatory state, such as IL1B, IL8, and IL23p19, that were induced by RD1 irrespective of the BCG or Mtb backgrounds (Fig. 2A), whereas the expression of the IL27p28 subunit, IFNB1, IL29 and the related ISGs were stimulated only when DC were infected with Mtb carrying RD1 (Fig. 2B). This result is reminiscent of global transcriptional profiling performed in tissues derived from mice infected with either wt or ESX-1 mutant bacilli or *in vitro* infected macrophages, where host ESX-1 controlled genes were predominantly IFN-regulated^32^. Additional genes and pathways were modulated by infection with Mtb and BCG recombinant strains, and further molecular and functional characterization is required to understand their contribution to immune response driven by infected DC.

Transcriptional profiling data showing the dependence of IL1B and IL23p19 expression on RD1 encoded factors were confirmed at the protein level; IL-1β and IL-23 were found in the supernatants of DC cultures infected with BCG or Mtb strains carrying the RD1 locus. TNF-α release was also induced in DC infected with both Mtb and BCG strains carrying the RD1 genetic locus. Conversely, the expression of IL-12 was strictly dependent on Mtb, even if deleted of RD1, while was absent in response to the infection with all BCG strains. The expression of IFN-β paralleled that of IL-12 since IFNB1 transcription was consistently induced upon DC challenge with Mtb clones and particularly with MtbΔRD1::RD1, whereas a weak transcriptional response was observed in response to all BCG recombinants irrespective of RD1 presence (our unpublished data).

This transcriptional profile well correlates with the activation of cellular signaling cascades, involving NF-kB and MAPK^26^,^33^,^34^. In particular, the activation of these pathways mainly induced in Mtb- rather than BCG-infected DC was further amplified when the RD1 locus was present, thereby enhancing the expression of pro-inflammatory cytokines, such as TNF-α and IL-23. In addition, the data on IRF3 modulation after the infection with wt Mtb and MtbΔRD1::RD1 are reminiscent of our previous findings showing that the transcription of IL12A (also known as IL12p35) subunit and of IFN-β1 via IRF3 activation takes place only in Mtb-infected DC, while is poorly induced in BCG-stimulated cells, which are consequently less efficient in promoting a Th1-oriented response^33^,^35^.

Within this context, it is important to reconsider recent data showing the ability of Mtb-induced IFN-β to restrict IL-1β synthesis in infected macrophages by limiting both IL1B transcription^36^ and AIM2 inflammasome activation^37^. Indeed, in MtbΔRD1::RD1-infected DC IL-1β production was similar or even higher than the levels found in BCG::RD1 challenged cells where IFN-β production was absent, suggesting that it is unlikely that Mtb exploits IFN-β to dampen IL-1β mediated inflammation, at least in human DC.

These results imply that different intracellular cytokine pathways may be activated depending on the presence or absence of RD1-encoded ESX-1 secretion system which, by acting on phagosomal permeabilization, may allow several mycobacterial factors to stimulate different intracellular signaling components, such as the DNA sensor IFI16/IFI204, TANK-binding kinase 1 (TBK)/IRF3, NLR family pyrin domain containing 3 (NLRP3) and caspase-1^32^,^38^,^39^. This interesting aspect of Mtb-host interaction requires further in-depth analysis to identify mycobacterial components and/or mycobacterial inducible mechanisms that fine-tune the balance between pro- and anti-inflammatory cytokines^40^.

Together with the modulation of the extra-cellular cytokine milieu, which acts both locally and systemically on multiple target cells to tune host immune response against Mtb^40^, the acquisition of a mature phenotype, required for an efficient antigen presentation, is a crucial event of pathogen defense signaling occurring in DC to instruct T cell response. Concerning this aspect, we observed that RD1 was inefficient in promoting full DC maturation when introduced in the BCG background, as compared to the response induced by wt Mtb infection. Similarly, no apoptosis was observed in DC in response to the infection with BCG strains carrying or not the RD1 region. In the case of DC infection with Mtb recombinants, however, a strong apoptotic phenotype was found induced by wt Mtb and the reconstituted strain MtbΔRD1::RD1, in which the RD1 locus was reintroduced. Nonetheless, MtbΔRD1::RD1-ESAT6Δ84-95 and MtbΔRD1::RD1-ΔpromCFP10 strains, both lacking the expression of a functional ESAT-6/CFP10 complex, promoted DC apoptosis although at different degrees, whereas infection with MtbΔRD1::B412 did not.

These interesting piece of data revealed on one hand that in BCG background the RD1 reintroduction is not sufficient to properly stimulate an apoptotic phenotype in infected DC. On the other hand, our results showed also that when the RD1 locus was present in a Mtb backbone, even if partially mutated leading to an non-functional ESX1 activity (as for both MtbΔRD1::RD1-ΔpromCFP10 and MtbΔRD1::RD1-ESAT6Δ84-95 strains), apoptosis will still occur. This well fits with a hypothesis in which other Mtb strain specific factors cooperate with RD1-encoded proteins (not only ESAT-6 and CFP10) for cell death induction. In accordance, recent findings obtained in mouse macrophages suggested that Mtb virulence influences the level of host cell death, while the balance of apoptosis and necrosis is controlled by other strain-specific characteristics^41^.

Among the Mtb strain specific factors it is important to acknowledge the influence of the three-gene operon containing espACD, whose regulation mediated by the two-component virulence regulation system PhoP/PhoR regulon, may differ in some members of the Mtb complex. Three mutations affecting the PhoPR genes, impair the production of molecules—including ESAT-6—involved in pathogenicity of animal-adapted lineages, as *Mycobacterium bovis*^42^. Nevertheless, these strains acquired compensatory mutations—as the deletion of the region upstream espACD operon, namely RD8—that short-cut the PhoP and EspR-enhancer like sequence-dependent regulation of espACD operon, thus restoring ESAT-6 secretion^42^.

Nonetheless, it has been also described that the deletion of the RD8 region, which mainly occurs in the majority of nonpathogenic species, may cause a lowered ESX-1 activity, since the secretory function of this system is dependent on the EspA and EspC mycobacterial proteins^43^. Providing that the RD1-encoded ESX1 locus is present in both pathogenic and nonpathogenic species, it is conceivable that ESX-1 per se does not play a direct role but it may be a prerequisite for Mtb virulence while EspA represents a major determinant of ESX-1-mediated virulence^43^. Based on these observations, it is likely that the short EspR-enhancer like sequence present in BCG^44^ may limit the expression of ESAT-6 and CFP-10 heterodimer in BCG::RD1 recombinant as compared to that present in wt Mtb, thus contributing to a specific host-pathogen interaction and leading to a defined cytokine profile, a poor DC maturation and a reduced apoptotic phenotype of infected DC.

Another important issue, emerging from this comparative analysis is that all Mtb recombinants possess the capacity to expand a Th1 population producing IFN-γ, which may be dependent on both the cytokine milieu released by Mtb-infected DC—IL-12, IFN-β and IL-23—and the Mtb wide antigen portfolio. Among Mtb specific antigens, the RD1-encoded factors significantly enhanced the capacity of BCG to stimulate in DC a Th1-promoting phenotype. The expansion of an IFN-γ producing Th1 population still represents an important correlate of vaccine-induced immunity – although recently debated^45^,^46^—since IFN-γ production potentiates macrophage killing, enhances phagosome maturation and increases lysosomal delivery of mycobacteria by activating autophagy. In addition, the extreme susceptibility of individuals bearing deficiencies in the IFN-γ and IL-12 pathways to develop TB^47^ argues that IFN-γ production by CD4^+^ T cells serves as a protective immune marker.

In conclusion, the results of this study further highlight the importance of examining the interaction of Mtb with human DC to provide important pre-clinical information about the immunogenicity of potential vaccine candidates. The ability of RD1-encoded factors to elicit different nuances of DC response depending on the BCG or Mtb backgrounds where they are inserted, sheds light on the existence of a fine interplay between host- and bacterial-regulated events controlling immune response, whose understanding would open new perspectives in therapeutic and vaccine strategies against Mtb.

## Methods

### Antibodies (Abs) and other reagents

Monoclonal Abs specific for CD1a, CD14, CD38, CD86, CD83, HLA-DR, IgG1, IgG2a (BD Bioscience), Annexin-V (Abcam) and FvDye eFluor R780 (eBioscience) were used as direct conjugates to Fluorescein isothiocyanate (FITC), Phycoerythrin or Allophycocyanin-7 as needed.

### DC preparation and infection

Istituto Superiore di Sanità Review Board approved the present research project (CE/13/387). DC were prepared as previously described^35^. Briefly, DC were generated by culturing CD14^+^monocytes with GM-CSF (50 ng/ml) and IL-4 (1000 U/ml) (R&D Systems) for 5 days at 0.5 × 10^6^ cells/ml in RPMI 1640 (Lonza), supplemented with L-glutamine (2 mM) and 15% Fetal Bovine Serum (FBS) (Lonza). At day 5 the cells were tested for their differentiation status by evaluating CD1a expression (>90% CD1a^+^) and lack of CD14 (>95% CD14^−^). Before infection, the medium was replaced with RPMI without antibiotics and supplemented with L-glutamine (2 mM) and 15% FBS. Cytokine deprivation did not affect DC survival rate, which was >90%.

DC cultures were then infected with a multiplicity of infection (MOI) of 1 bacteria/cell as previously described^31^ and internalization assays demonstrated that all different recombinant strains used in this study were taken-up by DC similarly to the parental Mtb (Supplementary Fig. S2).

### Bacteria description and preparation

Mycobacterial strains used in this study are listed in Supplementary Table S1. In particular, all Mtb recombinants were engineered from the mutant strain Mtb H37RvΔRD1 (MtbΔRD1)^4^ containing the deletion of the RD1 region^48^. All BCG recombinants were engineered from BCG Pasteur^49^. The pRD1-2F9 cosmid^6^,^7^, carrying the extended RD1 locus, was integrated into H37Rv∆RD1 strain^4^ and BCG to generate Mtb∆RD1::RD1 and BCG::RD1 strains while the control constructs MtbΔRD1::B412 and BCG::B412 contained the empty vector pYUB412.

For construction of Mtb- and BCG- ESAT6∆84-85 expressing strains, a modified version of the pRD1-2F9 cosmid harboring the last 12 codons of the ESAT-6-encoding gene replaced by a SpeI restriction site was used^6^. For Mtb∆RD1::RD1-∆promCFP10, a pRD1-2F9 cosmid variant, carrying a 122-bp in frame deletion immediately upstream the CFP-10-encoding gene, was employed^6^. Finally, the BCG::RD1-ESAT6G45T was obtained by using a pRD1-2F9 variant, into which a point mutation (G to T) had been introduced into the ESAT-6-encoding gene *esxA*, resulting in the substitution of the Gly^45^ residue with a Thr residue in the modified ESAT-6 protein^50^.

For each recombinant strain, a qualitative evaluation of ESAT-6 expression and secretion has been conducted on bacterial cell lysate and culture filtrate by western blotting (see below for details) to ensure the stability of the desired mutations (Supplementary Fig. S3).

Mtb H37Rv (ATCC 27294; American Type Culture Collection) was grown, as previously described^35^. Recombinant strains Mtb∆RD1::RD1, Mtb∆RD1::B412, Mtb∆RD1::RD1-∆promCFP10, Mtb∆RD1::RD1-ESAT6∆84-95, BCG::RD1, BCG::B412, BCG::RD1-ESAT6∆84-95 and BCG::RD1-ESAT6G45T were grown as specified above with the addition of hygromicin (50 μg/ml) (Sigma) as selection^6^,^50^,^51^,^52^.

Logarithmically growing cultures were centrifuged at 800 rpm for 10 min to eliminate clumped mycobacteria and then washed three times in RPMI 1640. Mycobacteria were re-suspended in RPMI 1640 containing 15% FBS and then stored at −80 °C until use. Bacterial frozen vials were thawed and bacterial viability was determined by counting the number of colony forming units (CFU). All bacterial preparations were tested for endotoxin contamination (<1 Endotoxin U/ml) by the Limulus lysate assay (Lonza).

### CFU assay

DC were infected with recombinant Mtb and BCG strains at MOI 1. Five h after infection, cell cultures were extensively washed with RPMI 1640. DC were then centrifuged at 150 × g for 10 min to selectively spin down cells while extracellular bacteria remained in the supernatants. Cells were lysed and CFU enumerated as previously described^31^.

### ESAT-6 expression

Parental and recombinant mycobacterial strains were grown in 20 ml of Sauton liquid medium at 37 °C for 6 days. Bacteria were harvested by centrifugation and culture filtrates, recovered after filtration through 0.2 μm pore-size filters, were concentrated using a filter with a 3-kDa cutoff (Centricon; Millipore). The mycobacterial pellets were washed twice and suspended in Tris 20 mM (pH 7.5) containing protease inhibitors (Roche Diagnostics GmbH). Cells were broken by shaking with 106-μm acid washed-glass beads (Sigma) for 8 min at speed 30 in a tissue lyser apparatus (Qiagen). The whole-cell lysates were then centrifuged at 14,000 rpm for 30 min to remove bacterial cell debris.

Bacterial supernatant and whole-cell lysate were separated on a 15% PAGE Bis-Tris gel and electroblotted onto nitrocellulose membranes (GE Healthcare, Freiburg, Germany). Immunoblot analysis was carried out with mouse ESAT-6 monoclonal Abs (Hyb 76-8; Statens Serum Institute, Copenhagen, Denmark) and detection achieved using horseradish peroxidase-conjugate anti-mouse secondary Abs. Finally, blots were visualized with Enhanced Chemiluminescence plus kit (GE Healthcare).

Once concentrated, bacterial supernatant were separated on a 15% PAGE Bis-Tris gel and electroblotted onto nitrocellulose membranes (GE Healthcare, Freiburg, Germany). Immunoblot analysis was carried out with mouse ESAT-6 monoclonal Abs (Hyb 76-8; Statens Serum Institute, Copenhagen, Denmark) and detection achieved using horseradish peroxidase-conjugate anti-mouse secondary Abs. Finally, blots were visualized with Enhanced Chemiluminescence plus kit (GE Healthcare).

### RNA extraction and Real-time PCR quantification

RNA was extracted from control or 8 h-infected DC, obtained from 4 independent healthy donors, with RNeasy kit (Qiagen Inc.) according to the manufacturer’s instructions. A phenol/chloroform extraction was performed to inactivate residual mycobacterial particles. Reverse transcriptions and quantitative PCR assays were performed as previously described^33^. The primers used for IFNB1, IL29, IL23p19, IL27p28, IL12p40, EBI3 and GAPDH were previously described^53^,^54^. Transcript expression was normalized to the GAPDH level using the Equation 2^−ΔCt^; the values are means ± SD of triplicate determinations.

### Transcriptomic analysis

DC cultures from 2 healthy donors were infected for 8 h with parental Mtb and the indicated Mtb and BCG recombinants. RNAs were extracted with mirVana isolation kit (Ambion, Life Technologies) according to the manufacturer’s instructions. RNA quantification and integrity was inspected by bioanalyzer analysis (Agilent Technologies). cRNAs were generated and hybridized on HumanHT-12 v4 Expression BeadChips according to the Illumina TotalPrep RNA Amplification Protocol (Illumina). The chips were then scanned with the Illumina scanner to generate the digitized image data files. Data were analyzed using the BeadStudio software package to generate a probe-level profile without background subtraction. Data were further analyzed with oneChannelGUI bioconductor package^17^. Specifically data were normalized by loess^55^ and the number of genes evaluated was reduced by applying an interquartile filter (IQR >0.25) to remove the non significant probe sets (i.e. not expressed and those not changing)^56^ (http://www.bepress.com/bioconductor/). To assess differential expression, we used an empirical Bayes method^57^ together with a false discovery rate correction of the p-value^58^. Hierarchical clustering (ST, Euclidean distance, average clustering) was performed using TMEV 4.8 software (http://www.tigr.org/software/). The genes annotated in the Ingenuity knowledge database were analyzed using the data-mining tool IPA 7.0 (www.ingenuity.com). Each treatment was subjected to IPA 4.0 analysis, and subsequently, the results were subjected to a comparative analysis to identify functional classes associated with differentially expressed genes in response to different treatments. Gene expression data have been deposited in the NCBI’s Gene Expression Omnibus (GEO, http: http://www.ncbi.nlm.nih.gov/geo, GEO Series accession number GSE62423), according to the MIAME standards.

### Flow cytometry analysis

Cells (10^5^) were washed once in PBS containing 2% FBS and incubated with monoclonal Abs at 4 °C for 30 min. After washing the cells were fixed in 4% formaldehyde (PanreacQuimica) before analysis on a FACSCan using CellQuest software (Becton Dickinson). A total of 20,000 cells were analyzed per sample. The viable DC were gated by forward and side scatter and then the expression of cell surface molecules was evaluated using the median fluorescence intensity (MFI) after subtraction of the values of the isotype Ab controls. Only cells comprised in viable cell gate were considered for the analysis.

### Immunoblot analysis

Western blots were performed as previously described^33^. Briefly, 25 μg of total cell extracts were separated by 10% SDS-PAGE gel and blotted onto nitrocellulose membranes.

Blots were incubated with rabbit polyclonal anti-IRF3 and rabbit polyclonal anti-NFkB p65 (Santa Cruz Biotechnology, Santa Cruz, CA), rabbit polyclonal anti-phospho-NFkB p65 (Ser536), rabbit polyclonal anti-p44/42 MAPK, rabbit polyclonal anti-phospho-p44/42 MAPK (Thr202/Tyr204), rabbit polyclonal anti-phospho-p38 MAPK (Thr180/Tyr182) (Cell Signaling Technology, Danvers, MA) and mouse monoclonal anti-p38 MAPK (R&D Systems, Minneapolis, MN) Abs. Detection was achieved using horseradish peroxidase-conjugate secondary Abs anti-mouse and anti-rabbit (Jackson Immunoresearch) and visualised with Enhanced Chemiluminescence plus (GE Healthcare). A ChemiDoc XRS (Bio-Rad Laboratories, Hercules, CA) was used to reveal the chemiluminescence.

### Cytokine determination

Supernatants of DC cultures were harvested 24 h after infection, filtered (0.2 μm) and stored at −80 °C. The production of IL-12, TNF-α and IL-1β was then measured by human Inflammatory Cytokine kit (Cytometric Bead Array; BD Bioscience). IL-23 and IFN-γ release was instead assayed by ELISA (R&D Systems).

### Apoptosis detection

Phosphatidylserine exposure and membrane integrity were analyzed by using Annexin V-FITC and FvDye according to manufacturing protocols. Twenty-four h post infection, DC were stained with FvDye and then labeled for 15 min with 5X Annexin V-FITC, as previously described^31^. Finally, the cells were fixed with formaldehyde 8% for 30 min before analysis on a FACSCanto (BD Bioscience). A total of 30,000 cells were analyzed per sample. Data were analyzed by FlowJo software (TreeStar Inc., Ashland, OR).

### MLR experiment

MLR was conducted as previously described^33^. Briefly, CD4^+^ T cells were purified by indirect magnetic sorting with a CD4^+^ T-cell isolation kit (Miltenyi) from allogeneic PBMC. Immature DC and DC infected for 24 h with wild type Mtb or Mtb and BCG recombinant strains described previously were re-suspended in 10% FBS complete medium prior to co-culture with total CD4^+^ T cells. Supernatants from T cell/DC co-culture (ratio 10:1) were harvested at day 5 and analyzed for IFN-γ production.

### Statistical analysis

Statistical analysis was calculated using a two-tailed Student’s t-test for paired data. A *p* value < 0.05 was considered statistically significant.

## Additional Information

**How to cite this article**: Etna, M. P. *et al.* Impact of *Mycobacterium tuberculosis* RD1-locus on human primary dendritic cell immune functions. *Sci. Rep.* **5**, 17078; doi: 10.1038/srep17078 (2015).

## Supplementary Material

Supplementary Information

## Figures and Tables

**Table 1:**
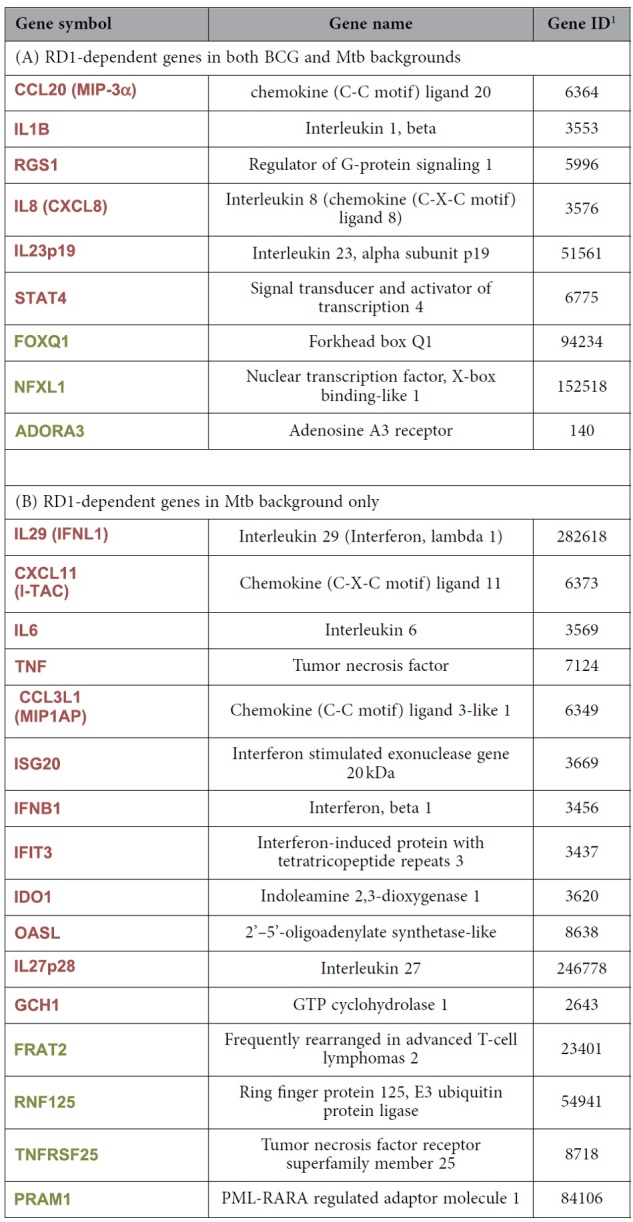
Selected top up- and down-regulated genes.

^1^http://www.ncbi.nlm.nih.gov/gene/ Selected top up- (in red) and down-regulated (in green) genes showing RD1 dependence in both Mtb and BCG backgrounds. (A) Selected top up- (in red) and down-regulated (in green) genes showing RD1 dependence in Mtb background only (B).
