# Supplementary material for: Impact of Mycobacterium tuberculosis RD1-locus on human primary dendritic cell immune functions
Source: Sci Rep. 2015 Nov 25;5:17078. doi: 10.1038/srep17078 (PMC4658526; doi:10.1038/srep17078)
Supplement: Supplementary Information [file srep17078-s1.doc]

**Impact of *Mycobacterium tuberculosis* RD1-locus on human primary dendritic cell immune functions**

Marilena P. Etna^1^, Elena Giacomini^1^, Manuela Pardini^1^, Martina Severa^1^, Daria Bottai^2,3^, Melania Cruciani^1^, Fabiana Rizzo^1^, Raffaele Calogero^4^, Roland Brosch^3^, Eliana M Coccia^1*^.

^1^Department of Infectious, Parasitic and Immune-mediated Diseases; Istituto Superiore di Sanità; Rome, Italy

^2^Department of Translational Research and new Technologies in Medicine and Surgery, University of Pisa; Pisa, Italy;

^3^Institut Pasteur, Unit for Integrated Mycobacterial Pathogenomics; Paris, France

^4^Molecular Biotechnology Center, Department of Molecular Biotechnology and Health Sciences; University of Torino; Turin, Italy

* Corresponding author: [eliana.coccia@iss.it](mailto:eliana.coccia@iss.it)

**Supplementary Figure S1. Impact of RD1 locus on the expression of IL12p40 and EBI3 subunits**


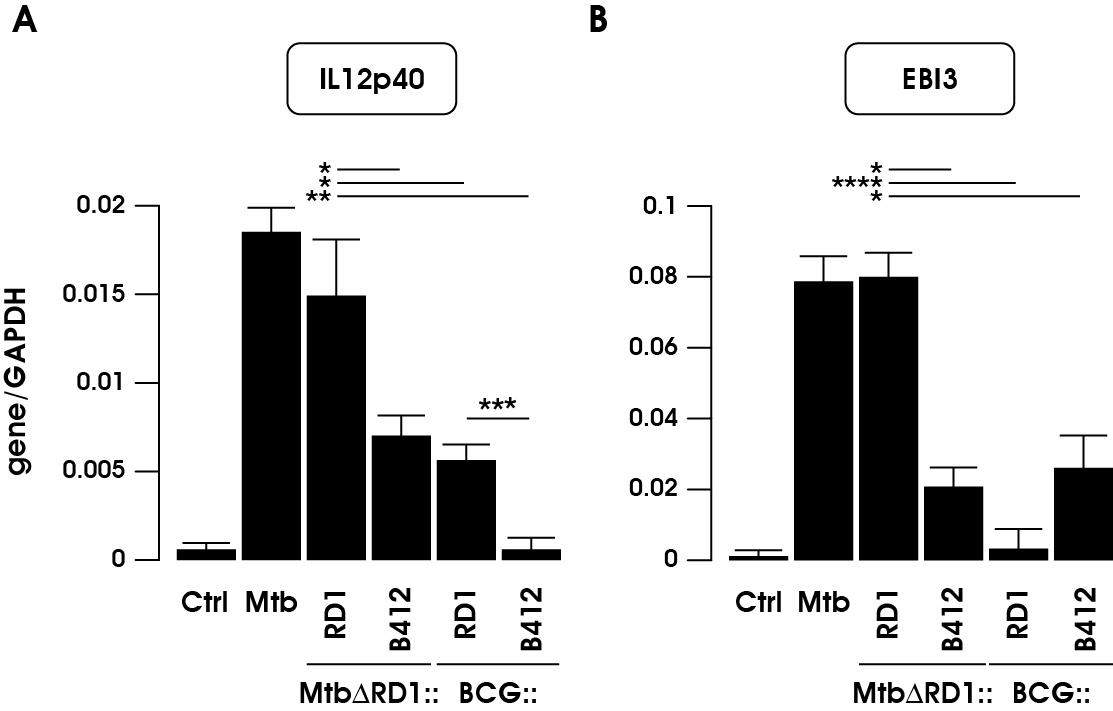


DC were left untreated (Ctrl) or infected with the indicated Mtb and BCG RD1-recombinant strains for 8 h. The expression of IL12p40 (A) and EBI3 (B) subunits was analyzed by real-time RT-PCR. Quantification data are normalized to the GAPDH level using the equation 2^-ΔCt^. The results shown are the means of 4 experiments performed with RNA extracted from DC cultures independent from those used in the transcriptome studies. p-values: * ≤0.05; ** ≤0.03; *** ≤0.015; **** ≤0.04.

**Supplementary Figure S2. DC internalize RD1-based BCG and Mtb recombinant strains at similar extent**


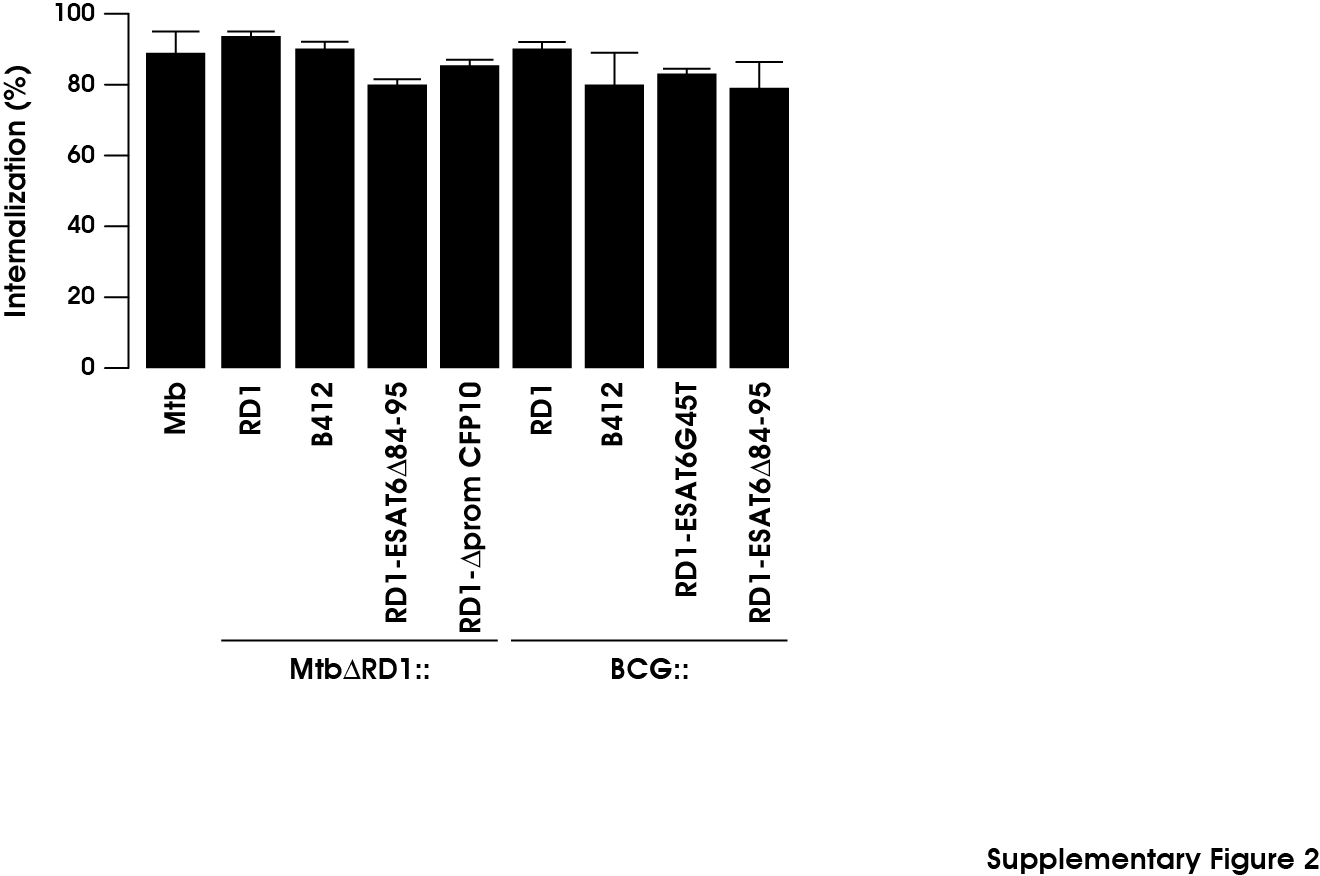


DC were infected for 5 h with parental Mtb and recombinant Mtb and BCG strains. DC were extensively washed and lysed to determine the number of internalized bacteria by CFU counting upon 21 days of culture. The results are presented as the percentage of internalized bacteria after 5 h in respect of the initial infective dose (0 h). The values are the means ± SD of 5 independent experiments.

**Supplementary Figure S3. Expression and secretion of ESAT-6 from Mtb and BCG recombinant strains used in the study**


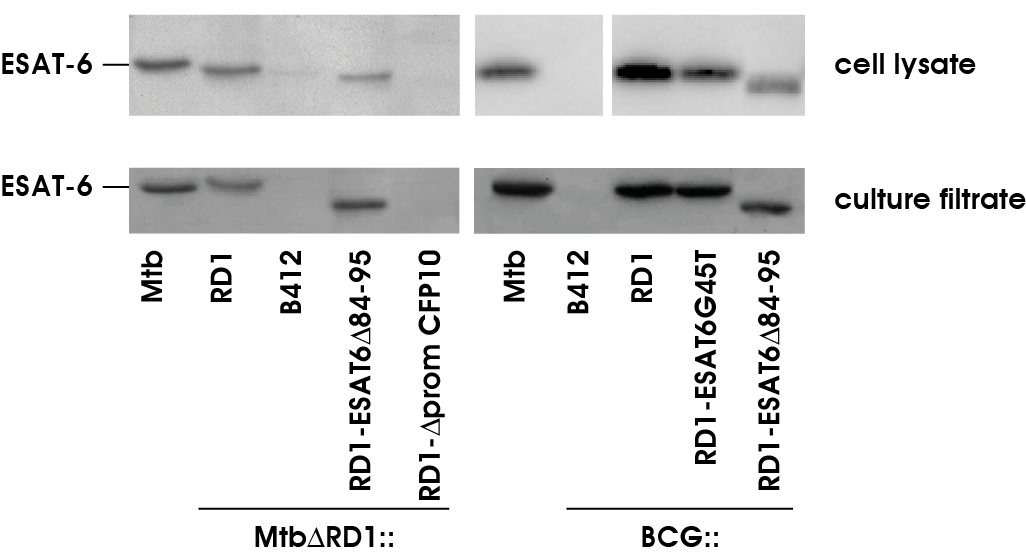


ESAT-6 expression and secretion was investigated by immunoblotting in cell lysates and culture supernatants from parental Mtb or Mtb and BCG mutant strains engineered in the RD1 genomic region. The result shown is from 1 out of 3 independent experiments that yielded similar results.

Supplementary Table S1. RD1-linked Mtb and BCG recombinant strains used in the study

| Name | Characteristic and ESAT-6 expression/secretion* |
| --- | --- |
| MtbΔRD1::RD1 ^50^ | The pRD1-2F9 vector, carrying the extended RD1 region form Mtb, was integrated into the MtbΔRD1 recombinant strain.  Secretion of a functional ESAT-6/CFP-10 heterodimer. |
| MtbΔRD1::B412 ^50^ | The pYUB412 empty vector was inserted in the MtbΔRD1 recombinat strain.  No expression of ESAT-6/CFP-10 heterodimer |
| MtbΔRD1::RD1-ESAT6Δ84-95 ^6^ | The pRD1-2F9 vector, carrying a truncated C-terminus variant of ESAT-6, was integrated in the MtbΔRD1 recombinant.  Secretion of 12 amino acid truncated ESAT-6 with CFP-10. |
| MtbΔRD1::RD1-ΔpromCFP10 ^6^ | The pRD1-2F9 vector, carrying the deletion of the CFP-10 promoter region, was integrated in the MtbΔRD1 recombinant strain.  No expression of ESAT-6/CFP-10 heterodimer. |
| BCG::RD1 ^49^ | The pRD1-2F9 vector, carrying the extended RD1 region from Mtb, was integrated into BCG.  Secretion of a functional ESAT-6/CFP-10 heterodimer. |
| BCG::B412 ^49^ | The pYUB412 empty vector was inserted into BCG.  No expression of ESAT-6/CFP-10 heterodimer. |
| BCG::RD1-ESAT6Δ84-95 ^6^ | The pRD1-2F9 vector, carrying a truncated C-terminus variant of ESAT-6, was integrated into BCG.  Secretion of 12 amino acid-truncated ESAT-6 together with CFP-10. |
| BCG::RD1-ESAT6G45T ^51^ | The pRD1-2F9 vector, carrying a amino acid substitution G to T at 45 position of ESAT-6, was integrated into BCG.  Secretion of a point-mutated ESAT-6 with CFP-10. |

* evaluated by western blot (see Supplementary Figure S3)

Description and ESAT-6 expression/secretion of RD1-recombinant Mtb and BCG strains used in the study. Numbers in the brackets refer to bibliography references where strains were firstly described.
